# The role of maternal care in borderline personality disorder and dependent life stress

**DOI:** 10.1186/s40479-018-0083-y

**Published:** 2018-03-20

**Authors:** Ericka Ball Cooper, Amanda Venta, Carla Sharp

**Affiliations:** 1 Department of Psychology & Philosophy, Sam Houston State University, Campus Box 2447, Huntsville, 77341-2447 TX USA; 2 Department of Psychology, University of Houston, Houston, TX USA

**Keywords:** Maternal care, Borderline personality disorder, Dependent life stress, Adolescents

## Abstract

**Background:**

Borderline Personality Disorder (BPD) affects 0.9%–3.2% of adolescents, and more than 20% of inpatient adolescents. Life stress has been linked to BPD across the lifespan, and previous research in adults has linked BPD to dependent stress (i.e., stress induced by the individual). However, prior research has not examined dependent stress alongside BPD in adolescents. Additionally, the potential protective effect of maternal care has not been considered in this relation. This study tested a moderation model expecting that (1) BPD would be positively associated with dependent life stress, (2) maternal care would be negatively associated with BPD, and (3) maternal care would moderate the relation between BPD and dependent life stress.

**Method:**

The sample consisted of 184 adolescents recruited from an inpatient psychiatric facility serving a diverse population in the Southwestern United States. Dependent life stress, BPD, and maternal care were measured using the UCLA Life Stress Interview, DSM-IV Childhood Interview for BPD, and Kerns Security Scale, respectively.

**Results:**

Results supported the first two hypotheses; BPD diagnosis was significantly, positively associated with dependent life stress, and negatively associated with maternal availability and dependability. Contrary to the third hypothesis, no significant evidence that maternal care acts as a buffer in the relation between BPD and dependent life stress was found.

**Conclusions:**

Although maternal care was not found to moderate the association between BPD and dependent life stress, results supported previously found relations between BPD, dependent life stress, and maternal care, and did so within a diverse inpatient adolescent sample.

Borderline Personality Disorder (BPD) is currently defined by the *Diagnostic Statistical Manual—5th edition* (*DSM*-5) as an emotion dysregulation disorder characterized by a consistent pattern of instability within several domains, including marked impulsivity in behavior, difficulty in interpersonal relationships, and frequent changes in mood or affect [1]. BPD has been found in 1.6% of the general population, and most studies estimate it affects one to 3% of the general population [1]. The development of BPD has been associated with both genetic and environmental factors in a variety of studies, providing support for the biosocial model [2]. This model suggests that the emotion dysregulation at the core of BPD is generated by a combination of biological and environmental factors [2, 3]. The broad aim of this study was to explore the interplay between two environmental factors—specifically the caregiving environment and stress, in relation to BPD symptoms among adolescent inpatients.

Stress has been demonstrated to confer risk for BPD through stressful events prior to diagnosis [4], and exacerbate BPD symptoms after diagnosis [5]. Critical to the current study, research suggests individuals with BPD uniquely contribute to their own experience of stress. As described in the stress generation hypothesis [6], dependent stress is induced or elicited by the individual rather than the situation, such as an adolescent with social anxiety avoiding social interactions, subsequently decreasing the quality of his interpersonal relationships. Indeed, DLS is more common in adults with BPD compared to patients with other personality disorders [6]. DLS may be a particularly relevant construct to study in adolescence, as stressful events occur more often in teens, and teens demonstrate increased sensitivity to stress [7]. Thus, the first aim of the current study was to explore the relation between DLS and BPD among adolescents.

A key variable to consider in the relation of stress and BPD is the caregiving environment, which is a significant feature in the development of BPD according to the biosocial model [8]. Specifically, in a normative adolescent sample, inconsistent maternal parenting style and maternal over-involvement were positively correlated with a later BPD diagnosis [9]. Within a sample of adolescent inpatients, maternal care has also been negatively associated with BPD diagnosis and symptom severity [10]. Finally, a plethora of maternal parenting qualities (e.g., adolescent-perceived maternal overprotection, maternal psychopathology, and parental absence) have all been linked with BPD symptom severity [11, 12]. These studies thus consistently demonstrate that low maternal care is a risk factor for BPD in adolescents [11–13]. For the present study, two specific aspects of adolescents’ perceptions of maternal care, modeled on Bowlby’s [14] theory, were considered: availability and dependability; specifically, the second aim of the present study was to explore the relation between BPD and maternal care among adolescents.

Because maternal care can also serve a protective function against psychopathology, it may be a significant moderating factor in the relation between stress and BPD. Indeed, positively perceived maternal care has been linked with decreased occurrences of DLS in a depressed population [15]. Additionally, attachment style to one’s maternal figure was found to moderate the association between severity of mental illness and dependent stressful life events in depressed adults [15], such that there was a significant, positive relation between these constructs among individuals with an insecure attachment, but not those with a secure attachment. The proposed mechanism by which this may occur is either through these individuals fearing closeness with another individual and intentionally generating stress, or perceiving interpersonal stress as satisfactory and rewarding [15]. This proposal has not been previously evaluated in adolescent BPD; thus, the third aim of the present study was to explore maternal care as a potential moderator of the relation between dependent stress and BPD.

The current study examined the relation between BPD and DLS in a sample of low-income adolescent inpatients, exploring how the perceived maternal care environment (i.e., availability, dependability) may moderate this association. We hypothesized that (1) BPD would be positively associated with DLS, (2) measures of maternal care would be negatively associated with BPD, and (3) measures of maternal care would moderate the relation between BPD and DLS, such that maternal care would act as a buffer against the effect of BPD on DLS. The existing literature has thus far not explored a moderation model wherein maternal care moderates the association between BPD and life stress. Indeed, understanding how perceptions of maternal care affect the relation between BPD and DLS could impact adolescents who have BPD, and identify those individuals whose caregiving environment may place them at higher risk of experiencing dependent stress.

## Method

### Participants

The aforementioned hypotheses were examined via archival data collected from 184 participants (see Table 1 for demographics) from a public inpatient adolescent psychiatric facility in a large metropolitan city in the Southwestern United States. Parental consent and youth assent were obtained prior to data collection. Inclusion criteria for this study were an age between 12 and 17 years old and English fluency. Adolescents were excluded from study participation if psychosis or intellectual disability was noted during the admission evaluation by clinicians, or if consent or assent was denied. The current study focused on a sample of inpatient adolescents who completed measures of BPD, maternal care, and DLS.

**Table 1:** Demographic Statistics

Study participants (*N* = 184)	
Age	14.72 (1.42) years
Gender	
Female	121 (65.8%)
Male	63 (34.2%)
Race/Ethnicity	
African American	41 (22.3%)
Caucasian	55 (29.9%)
Hispanic	73 (39.7%)
Southeast Asian	1 (0.5%)
Multiracial	11 (6.0%)
Other Race/Ethnicity	3 (1.6%)
CI-BPD Diagnostic Category	
Met Criteria for BPD	58 (31.5%)
Did not Meet Criteria for BPD	126 (68.5%)

### Measures

**Dependent life stress** was measured via the University of California Los Angeles Life Stress Interview’s (UCLA-LSI) dependent life stress subscale [16] and utilized the number of dependent life events that participants endorsed in the past three months. **Borderline diagnosis** was assessed with the Childhood Interview for BPD (CI-BPD) [17], in which a score of 5 or higher was indicative of a positive diagnosis. Finally, **maternal care** was measured via the availability and dependability subscales of the Kerns Security Scale (SS) [18, 19]. All measures demonstrated adequate to high internal consistency within the present sample.

### Procedures

This study examined archival data that was collected as part of a study approved by the relevant institutional review boards. Procedures for the initial study were as follows: consent was first obtained from patients’ guardians at the time of admission. If granted, assent was also sought from participants prior to any interviews taking place. Demographic information was acquired first, followed by the CI- BPD, SS, and UCLA-LSI. Adolescents were paid $30 in the form of a gift card for participating. All study procedures were conducted independently and in private by interviewers who had been trained on study measures, and received supervision from the study’s principal investigator. Consensus meetings for the UCLA-LSI were held after the assessment was complete.

## Results

Study aims one and two sought to examine relations between (a) BPD and DLS and (b) BPD and maternal care. Regarding the first hypothesis, a Spearman’s Rho correlation was conducted due to the dependent life events variable having a non-normal distribution (Shapiro-Wilk = .82, *p* < .001), and a significant positive correlation was found with BPD diagnosis, *r*_s_(184) = .14, *p* = .04. Regarding the relation between BPD and maternal care, significant group differences were found for both Availability, *t*(182) = 3.37, *p* = .001, and Dependability, *t*(182) = 2.87, *p* = .01, with lower mean scores in the BPD group.

Multivariate analyses were used to follow up on significant relations identified through bivariate analyses for aims one and two, as well as to examine study aim three, which sought to explore maternal care as a moderator of the relation between BPD and DLS. Due to the assumption of normality being violated for the number of dependent life events variable, Poisson and Negative Binomial generalized linear models were examined for model fit. The Poisson probability distribution demonstrated poor fit, Goodness of Fit: *Deviance* = 269.06, *df* = 176, *p* < .001; however, the Negative Binomial probability distribution had an adequate fit, Goodness of Fit: *Deviance* = 134.96, *df* = 176, *p* = .99. Thus, the Negative Binomial generalized linear model was chosen to test the main effect of BPD (dichotomous; see Table 2), the main effect of maternal availability (continuous), the main effect of maternal dependability (continuous), the interaction effect of BPD and availability (continuous), and the interaction effect of BPD and dependability (continuous) on the number of dependent life events (count). Sex (categorical) and race (categorical) were included as covariates in the model. The inclusion of these predictors was not a significant improvement over the intercept-only model, *Likelihood Ratio Chi-Square* = 5.24, *df* = 7, *p* = .63. Thus, individual main and interaction effects were not interpreted.

**Table 2:** Results of Negative Binomial generalized linear model with dependent stressful life events as dependent variable

Predictor Variable	*B (SE)*	*Wald Chi-Square*	*p*	Confidence Intervals
Sex	.133 (.230)	.335	.563	−.317–.583
Race/Ethnicity	.021 (.046)	.221	.638	−.068–.111
BPD Diagnosis	−.294 (.725)	.165	.685	−1.715 – 1.126
Availability	−.147 (.241)	1.578	.209	−.620–.326
Dependability	.129 (.282)	.914	.339	−.424–.682
BPD x Availability	−.107 (.310)	.120	.729	−.714–.500
BPD x Dependability	.084 (.359)	.055	.814	−.618–.787

*Note.* BPD Diagnosis was assessed dichotomously using the Childhood Interview for BPD (CI-BPD), and Availability and Dependability were assessed continuously using the Kerns’ Security Scale (SS). *df* = 1 for all variables

## Discussion

The present study aimed to examine the relation between BPD and DLS, as well as the potential moderating role of maternal care in adolescents. Regarding our first hypothesis, the number of dependent life events was significantly associated with adolescents’ diagnostic group. These findings are thus consistent with previous research showing that BPD is positively correlated with both the number of overall stressful life events [6, 20], and dependent-specific stressful life events [21, 22]. Furthermore, the current study extends those results to a sample of low-income inpatient adolescents, as previous studies have only examined adults in this capacity. Second, negative associations between BPD and both maternal availability and dependability were supported, thereby confirming our hypothesis, bolstering previous research [23], and extending it to an adolescent inpatient sample. Lastly, despite our expectation that maternal care would function as a buffer between BPD and DLS, this hypothesis was not supported, as the overall generalized linear models examining these variables were not significant improvements upon the intercept-only models. Thus, although maternal care has previously been identified as a protective factor attenuating the relation between psychopathology and dependent life stress [15], as well as life stress more broadly [24], no evidence of such a role was noted in the current study.

In sum, although the hypothesized effect of maternal care as a moderator of the relation between BPD and DLS was not supported, the positive association found between BPD and DLS may stand to inform interventions for adolescents with BPD, as the latter could be made a focal point of treatment. Similar treatment interventions have demonstrated promise among individuals with depression when attempting to reduce DLS, and are theorized to show greater utility within an adolescent population [25]. In addition, incorporating the adolescent’s parents or caregivers into the treatment intervention may still be useful by encouraging the caregivers’ psychoeducation of stress-generating behaviors and its high occurrence among patients with BPD. Therefore, the impact of the present study lies in identifying adolescent’s stress-causing behaviors and targeting them for the sake of intervention, with the aim of not only reducing the behaviors themselves, but also the correlates of DLS (e.g., increased number of BPD symptoms and suicidal ideations) previously established by the literature base.

## Abbreviations

APA: American psychiatric association
BPD: Borderline personality disorder
CI-BPD: Childhood interview for borderline personality disorder
DLS: Dependent life stress
*DSM-*5: *Diagnostic and statistical manual*-5th edition
SS: Kerns security scale
